# Self-assembled hydrophobin for producing water-soluble and membrane permeable fluorescent dye

**DOI:** 10.1038/srep23061

**Published:** 2016-03-15

**Authors:** Kunpeng Wang, Yunjie Xiao, Yanyan Wang, Yaqing Feng, Cheng Chen, Jie Zhang, Qian Zhang, Shuxian Meng, Zefang Wang, Haitao Yang

**Affiliations:** 1 School of Chemical Engineering and Technology, School of Life Sciences, College of Precision Instrument and Opto-electronics Engineering, Tianjin University, Tianjin 300072, People’s Republic of China; 2 State Key Laboratory of Medicinal Chemical Biology, College of Pharmacy, Nankai University, Tianjin 300071, People’s Republic of China; 3 Tianjin International Joint Academy of Biotechnology and Medicine, Tianjin 300457, People’s Republic of China; 4 Collaborative Innovation Center of Chemical Science and Engineering, Tianjin 300072, People’s Republic of China

## Abstract

Low water solubility and poor membrane permeability are major disadvantages that compromise applications of most fluorescent dyes. To resolve these problems, herein, using Boron-dipyrromethene (BODIPY) as a model fluorescent dye, for the first time, we provide a new strategy for the rapid and efficient production of a water-soluble and membrane-permeable dye by mixing with an amphiphilic protein named hydrophobin. Data shows BODIPY could be effectively solubilized and dispersed in 200 μg/mL hydrophobin by simple mixing and sonication. Subsequent experiments indicated that hydrophobin self-assembled into a protein film on the surface of BODIPY forming stable hydrophobin-BODIPY complexes with a size range of 10–30 nm. Furthermore, we demonstrated hydrophobin-functionalized BODIPY are toxicity free to cells. The hydrophobin-BODIPY complex could pass through both the cell plasma membrane and nuclear membrane efficiently. Our work opens a novel route to modify and functionalize fluorescent dyes and may be developed as a general strategy for broadening their applications.

Fluorescence imaging has nowadays become the most powerful and exciting technique to visualize and monitor specific targets or processes in living systems^1^,^2^. Naturally, fluorescent dyes or probes play key roles in constructing and developing various fluorescence-based analyses^3^,^4^. Therefore, they have been extensively investigated to improve their abilities of analytical specificity and sensitivity, as well as the spatial and temporal sampling. However, fluorescent dyes do suffer from several fundamental problems including low water solubility and poor membrane permeability when they were used for bio-labeling and bio-imaging^5^,^6^,^7^.

Great efforts have been made to overcome these disadvantages of fluorescent dyes. For example, various hydrophilic groups, such as sulfonate, pyridinium, glycol, and carboxylate, were appended to the core of dyes to increase their aqueous solubility^8^,^9^,^10^. Introduction of a functional group such as the ester or carboxylic acid into dyes could increase their membrane permeability^11^,^12^. Although the modification of existing dye skeletons with appropriate functional groups could improve their water solubility and membrane permeability to some extent, it led to several new issues or problems subsequently. For example, inevitable increase of molecular weights of dyes resulted in interference with the function of biomolecules, apart from synthetic challenges^13^,^14^. Moreover, large molecular weight dyes could not be readily used for biomolecules *in vivo*, such as amyloid labeling, since such studies required the penetration of blood-brain barrier^15^,^16^, and could cause increasing in the serum pharmacokinetics of drug-dye conjugates as well^17^,^18^. Therefore, new strategies are required to solve those problems without compromising applications of fluorescent probes. Hydrophobin are a novel type of amphiphilic small proteins produced by the filamentous fungi, which are characterized for their self-assembling into protein films on different hydrophobic/hydrophilic surfaces to modify their surface properties^19^. The ability of hydrophobin to convert hydrophobic surfaces to hydrophilic ones has been documented in several studies^20^,^21^,^22^,^23^,^24^. Considering the properties of hydrophobin, we proposed that hydrophobin might alter the hydrophobicity of fluorescent dyes to improve their abilities of solubility and membrane permeability.

To test our hypothesis, we chose boron-dipyrromethene (BODIPY) as the model fluorescent dye in our study. BODIPY now represents one of the most popular family of fluorescent dyes for fluorescence imaging due to their strong absorption with high extinction coefficient, high fluorescence quantum yield, narrow absorption and emission bands and excellent photostability^25^. Moreover, BODIPY dyes in NIR window (650–900 nm) are favorable for *in vivo* fluorescent bio-imaging because of their minimum photo-damage to biological samples, deep tissue penetration, and minimum interference from background auto-fluorescence by biomolecules in living systems^26^. However, BODIPY indeed suffer problems of low-water solubility and poor membrane permeability^27^,^28^.

In this paper, firstly we synthesized a new near infrared region BODIPY derivative that was characterized by ^1^H NMR and MALDI-TOF-MS spectra. Then we systemically investigated the interaction of this BODIPY derivative and hydrophobin HFBI from different aspects. Finally, we evaluated the water solubility, membrane permeability and cytotoxicity of this HFBI/BODIPY hybrid. Our results demonstrated BODIPY could be effectively solubilized and dispersed in as low as 200 μg/mL HFBI solution, and nontoxic HFBI-functionalized BODIPY could not only pass through the cell plasma membrane, but also the nuclear membrane efficiently. To our knowledge, this is the first demonstration of functionalization of fluorescent BODIPY dye with amphiphilic protein, indicating the great potential of modification and functionalization of chemical fluorescent dyes with biological molecules.

## Results

### Design, synthesis and characterization of a long-wavelength BODIPY dye

**Figure 1:**
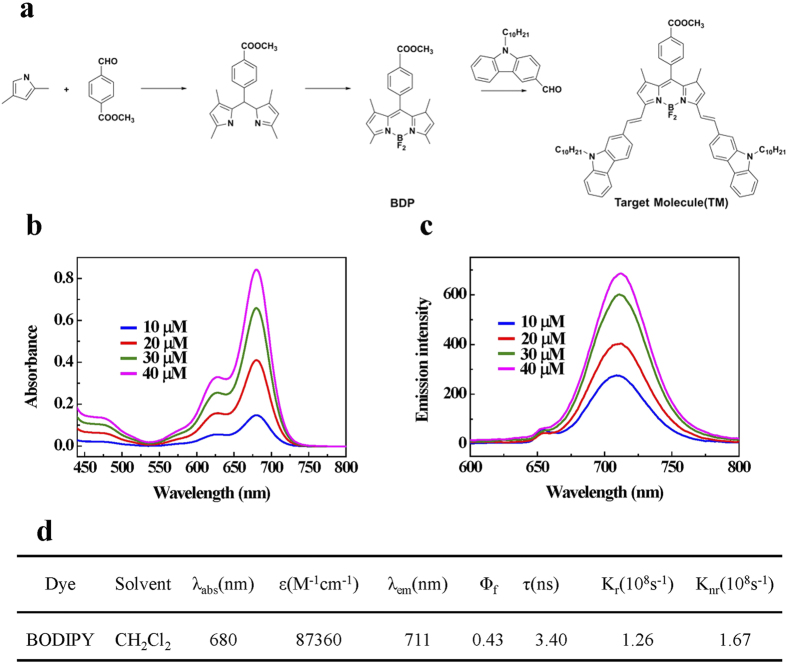
Synthesis and characterization of a long-wavelength BODIPY dye. **(a)** Synthetic procedure for the synthesis of the BODIPY derivative. **(b,c)** Absorption and fluorescence spectra of the BODIPY dye at different concentrations (10, 20, 30 and 40 μM) in CH_2_Cl_2_ respectively. **(d)** Photophysical properties of the BODIPY dye. The excitation wavelength was 660 nm in the fluorescence measurement.

In this study we firstly designed and synthesized a long-wavelength BODIPY dye that would be used in the following studies by the strategy of forming moderate intramolecular charge transfer (ICT) structures. The ICT strategy was relatively easy to accomplish the synthesis and was very effective in producing red shifts as well^29^. The design and synthesis of the BODIPY derivative was shown in Fig. 1a. Briefly, the carbazole derivative was introduced to the 3- and 5-methyl sites through the Knoevenagel reaction. And the decyl was introduced to BODIPY through carbazole to enhance its solubility in non-polar solvents, which facilitated preparation and characterization this derivative. With a few steps, the BODIPY dye was obtained with a reasonable yield that was about 46.5%, and was characterized by ^1^H NMR (Supplementary Fig. 1) and MALDI-TOF-MS (Supplementary Fig. 2) spectra. Since the intrinsic water-insolubility of this BODIPY dye, we investigated the absorption and emission spectra of the BODIPY dye in CH_2_Cl_2_ at different concentrations. As shown in Fig. 1b, the absorption spectra of this dye contained narrow spectral bands with two absorption peaks in the visible region. The intense band at 680 nm was attributed to the S_0_ − S_1_ transition, and the pronounced shoulder on the high-energy side of the main band, was resulted from the S_0_ − S_2_ vibrational transition^30^. This BODIPY derivative in CH_2_Cl_2_ shows an emission spectrum (Fig. 1c) with a maximum at 711 nm, and the small peak at 660 nm (excitation wavelength) could be Rayleigh and Tyndall scattering in the emission spectra. The photophysical data of the BODIPY dye was summarized in Fig. 1d.

### Solubilization of the BODIPY dye with hydrophobin HFBI

**Figure 2:**
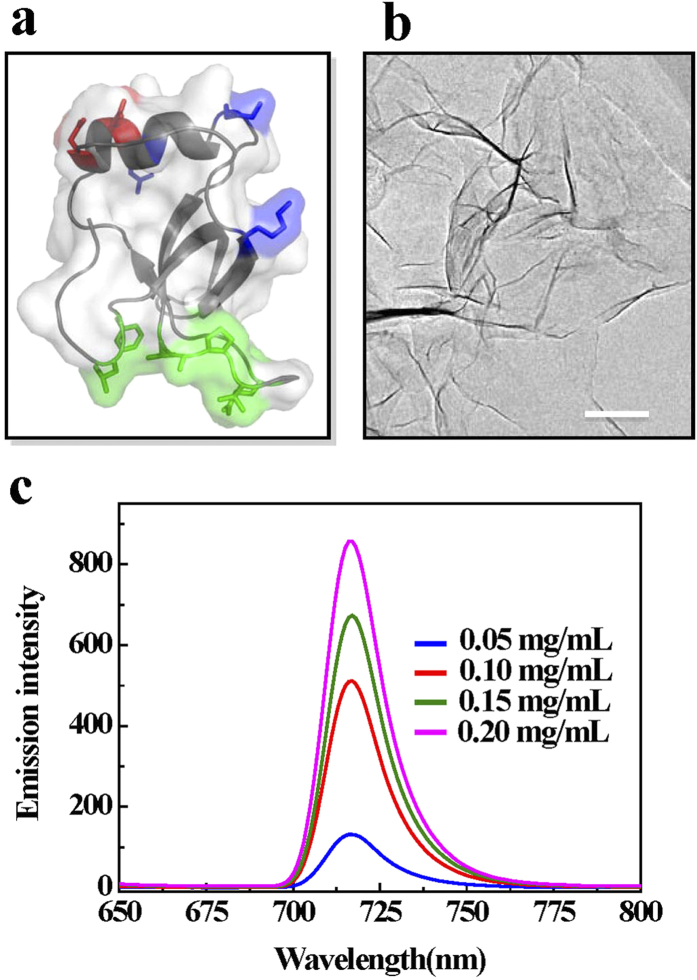
Hydrophobin HFBI can solubilize the BODIPY dye. **(a)** The amphiphilic structure of hydrophobin HFBI. The green part in the surface of HFBI is the hydrophobic patch that can bind to the hydrophobic solid surfaces. **(b)** The protein film formed by hydrophobin HFBI on the TEM copper grid. **(c)** The fluorescence spectra of the BODIPY dye solubilized at different concentrations (50, 100, 150, 200 μg/mL) of HFBI.

To overcome the solubility problem of the BODIPY dye, hydrophobin HFBI was employed to functionalize the hydrophobic BODIPY derivative in our study. As shown in Fig. 2a, HFBI is an amphiphilic protein with a large hydrophobic patch on its surface, which is accounted for about 18% of the total surface area^31^. This hydrophobic patch can directly interact and bind with different hydrophobic materials^32^. By this special structure, hydrophobin can assemble into a protein film at any interfaces, like solid-air, air-liquid, solid-liquid interfaces. Figure 2b shows a hydrophobin film formed on a TEM copper grid. It was also clearly observed that this kind of protein film could form wrinkles or folds that implied the flexibility of this film. Szilvay *et al*. also observed the occurrence of wrinkles in HFBI film at the air-water interface when the aqueous subphase was disturbed^33^. The amphiphilicity and flexibility of hydrophobin makes it an ideal and convenient “natural tool” to modify diverse hydrophobic targets with different shapes. Figure 2c shows the results of applying of different concentrations of hydrophobin HFBI to solubilize the BODIPY derivative. Overall, the intensity of fluorescence spectra of the BODIPY increased with the increasing of HFBI concentration. By increasing the protein concentration up to 200 μg/mL, this BODIPY derivative gave a very strong and sharp emission peak at 717 nm that was different from the one obtained in the CH_2_Cl_2_ solution. This result indicated that HFBI protein (200 μg/mL) could dissolve BODIPY effectively. When dissolved the BODIPY dye in 300 μg/mL HFBI, we found the emission intensity of the dye increased about 4.5% when compared with that obtained at the condition of 200 μg/mL HFBI (Supplementary Fig. 3a). However, hydrophobin-functionalized BODIPY dye was not stable and precipitated within three weeks at the condition of 300 μg/mL HFBI (Supplementary Fig. 3b). This result indicated that protein concentration was really a critical factor for the dispersion of the BODIPY dye. We had found this concentration-dependent manner before when we used hydrophobin to functionalize multi-walled carbon nanotubes^21^. The excessive free hydrophobin might work as “linkers” that connected the dispersed BODIPY dyes together strongly, making them form aggregates again. Nevertheless, we concluded that hydrophobin HFBI was served as an excellent solvent for the BODIPY probe. To our knowledge, this is the first demonstration by using protein to modify the BODIPY dye to increase its water-solubility.

### HFBI-functionalized BODIPY formed spherical particle in a HFBI protein concentration dependent manner

**Figure 3:**
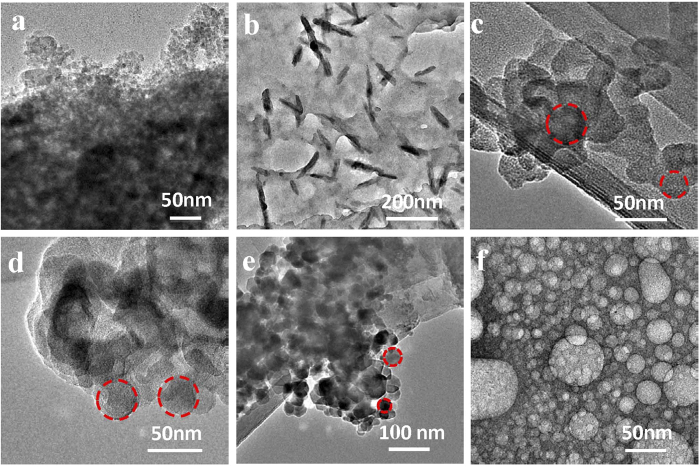
TEM images of BODIPY dissolved in different solvents. **(a)** BODIPY formed significant aggregation in water. **(b)** BODIPY was dissolved in methanol with typical rods like structures. **(c–f)** BODIPY was solubilized in HFBI protein solution at different concentrations respectively (50, 100, 150, 200 μg/mL). BODIPY dye was dispersed in HFBI solution in a protein-concentration dependent manner. At low HFBI concentrations (**c**–**e**), round shape particles (marked with red circles) could be observed although BODIPY dye was still aggregated. When protein concentration was increased to 200 μg/mL (**f**), all the BODIPY dye was dispersed and changed into round shape particles.

**Figure 4:**
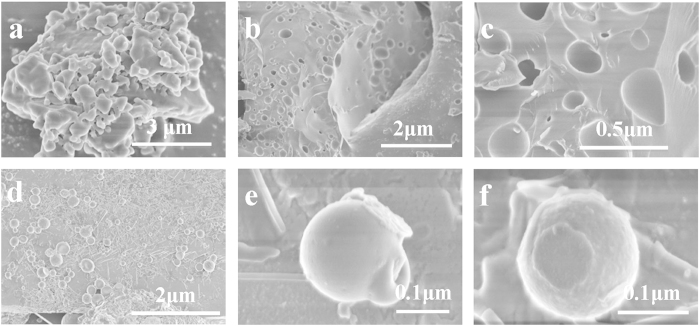
SEM images of BODIPY and HFBI/BODIPY complexes. **(a)** BODIPY dye aggregated with a polymorphous appearance. (**b,c**) Plentiful “small holes” presented on the BODIPY surfaces with different dimensions. Some holes were empty, but others were filled with HFBI/BODIPY complex. **(d)** The size of the HFBI/BODIPY complex was in the range of 10–200 nm. **(e)** Round HFBI/BODIPY complexes were budding from the BODIPY surface with various sizes. **(f)** A small piece of HFBI protein film was formed on the surface of HFBI/BODIPY complex.

The emission spectra of Fig. 2c gave us a clue that protein concentration might play an important role in the solubilization of BODIPY. To characterize the formation process of HFBI/BODIPY complex, we investigated morphological changes of the BODIPY dye dispersed at different HFBI concentrations. We used water and methanol as control solvents to make comparisons with HFBI during our measurements as well. Figure 3a shows significant aggregation of BODIPY dye in pure water indicating its natural property of low water solubility. However, typical rods like structures were observed with hundred nanometers when BODIPY dye was dissolved in pure methanol, suggesting reasonable solubility of BODIPY dye in methanol (Fig. 3b). When used HFBI to dissolve BODIPY dye, we found protein concentration did play a fundamental role during its dispersion processes. As shown in Fig. 3c, BODIPY dye aggregated when HFBI protein concentration was 50 μg/mL, but some spherical or round shape structures already formed on the surface of the BODIPY aggregate at this condition. When protein concentration increased, more and more spherical structures that were HFBI/BODIPY complexes could be found in Fig. 3d,e. When the protein concentration was up to 200 μg/mL, the BODIPY dye was turned into spherical shape particles that were dispersed very well. The sizes of HFBI/BODIPY complexes in Fig. 4f were quite homogeneous in the range of 10–30 nm. These results indicated that hydrophobin HFBI could dissolve and disperse BODIPY dye very efficiently at a rather low protein concentration. The detailed process for forming round HFBI/BODIPY complexes was unknown at this stage. We speculated that HFBI protein might work as an amphiphilic stabilizer onto the particle/water interface limited the crystal growth and restricted the shape of this BODIPY dye^23^.

### Spherical HFBI/BODIPY complexes were detached from huge BODIPY aggregates by making “small holes” on their surfaces

The TEM result shows BODIPY formed small round particles when solubilized in HFBI protein solution. Scanning electron microscopy (SEM) was then used to investigate more detailed process or possible mechanism about the formation of HFBI/BODIPY complexes. As shown in Fig. 4a, the hydrophobic BODIPY dye aggregated tremendously with a polymorphous appearance, which indicated that it was thermodynamically unstable and easily aggregated in high-polar solvent. Nonfluorescent aggregate of BODIPY dye was a realistic barrier for its biological and medical applications^34^. After application of hydrophobin HFBI, we found some very striking and interesting morphology changes of BODIPY dye. As shown in Fig. 4b, there were plenty “small holes” on the BODIPY surface with different dimensions. Some holes were empty, but others were filled with spherical shape particles that we believed they were HFBI/BODIPY complexes. The high magnification SEM image (Fig. 4c) showed more details about those small holes and particles. We could clearly see that a round HFBI/BODIPY complex was budding from the round holes on the BODIPY surface. The smallest hole in this picture was about dozens of nanometers. Figure 4d showed the size of the resulting HFBI/BODIPY complex was in the range of 10–200 nm, which was consistent with the TEM results. In Fig. 4e, the entire round BODIPY particle was covered HFBI film suggesting again the flexibility of this HFBI film^35^. Moreover, we could see a small HFBI/BODIPY particle was almost budding away from the big ones. From those results, we could conclude that the small HFBI/BODIPY particles we observed both in SEM and TEM were formed by detachment from the bigger ones during the sonication process. This phenomenon was quite different from the observation by other researcher who used HFBI to modify and dissolve graphene and carbon nanotubes^36^,^37^. Moreover, Fig. 4f showed a typical result that a small piece of HFBI protein film was attached onto the smooth surface of spherical particles, which indicated that at least HFBI monolayer was needed for the BODIPY dye to develop into a rather smooth sphere. The formation of multilayer films was likely due to charge interactions between HFBI proteins^38^.

### XPS and FI-TR measurements confirmed the physical adsorption of HFBI onto the BDDIPY surface

**Figure 5:**
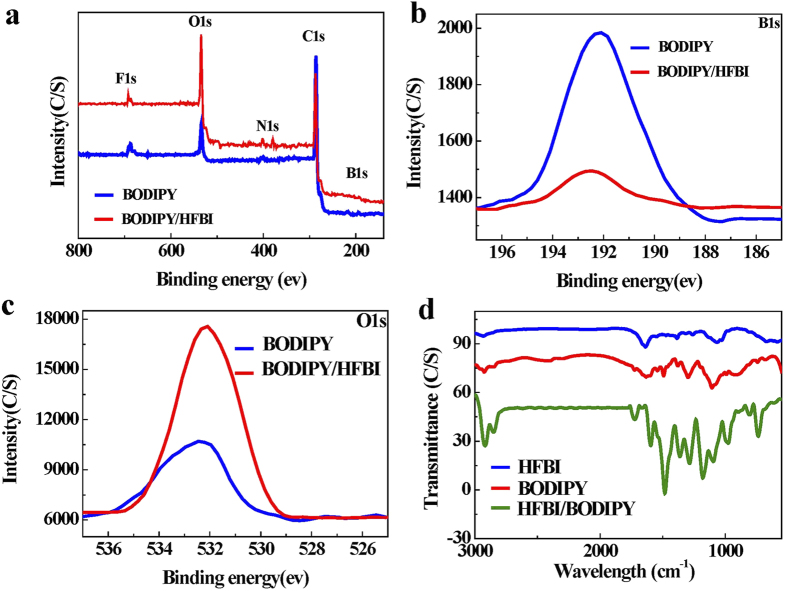
XPS and FI-TR measurements confirmed the physical adsorption of HFBI onto the BDDIPY surface. **(a)** Wide XPS spectra of BODIPY and HFBI/HGFI. **(b)** High-resolution spectra of B1s. **(c)** High-resolution spectra of O1s. **(d)** FT-IR spectra of HFBI, BODIPY and HFBI/BODIPY.

To confirm the absorption of HFBI on BODIPY, chemical compositions of BODIPY before and after HFBI modification were analyzed by X-ray photoelectron spectroscopy (XPS) respectively. The full XPS spectra (Fig. 5a) gave an overall change of the N1s, C1s, O1s, B1s and F1s of the BODIPY and HFBI-modified BODIPY. The B1s (191.3 eV) peak was characteristic for BODIPY dye according to its chemical compositions. This elemental peak was displayed but with decreased content in the narrow spectrum of HFBI-modification BODIPY (Fig. 5b), suggesting that HFBI protein was covering the surface of BODIPY. Furthermore, increased chemical content of oxygen (Fig. 5c) also confirmed the adsorption of HFBI protein on the surface of BODIPY. In the following, FI-TR (Fig. 5d) was used to characterize HFBI/BOPIPY due to the shape and frequency of the amide I band was very sensitive to the secondary structure of a protein. The FI-TR spectrum of HFBI protein alone showed characteristic peaks at 1685 and 1637 cm^−1^ related to the vibration bands of amide I, which were caused by C = O stretching vibrations of peptide linkages. This result indicated HFBI contained mainly beta-sheet (1637 cm^−1^) and random coil (1685 cm^−1^) structures. In the FI-TR spectrum of BODIPY, strong peaks at 2915 and 2850 cm^−1^ were arrtributed to the C-H vibrations of the benzene bonds, and peak at 1719 cm^−1^ was resulted from C = O vibration. Moreover, sharp peaks at 1574 and 1481 cm^−1^ corresponded to v (C = C) stretching vibrations in benzene. Regarding to the HFBI/BODIPY complex, the appearance of both characteristic peaks of HFBI and BOPIPY without novel chemical bonds formation suggested that HFBI adsorbed onto the surface of BODIPY dye non-covalently without protein structure change. This result was quite consistent with the previous reports. There was no secondary structure change during the adsorption process when class II hydrophobin assembled physically onto a hydrophobic surface^39^. The FI-TR result also strengthened the notion that physical hydrophobic force between the hydrophobic part of HFBI and BODIPY dye was the major driving force for their interaction and formation of HFBI/BODIPY complex.

### HFBI-functionalized BODIPY passed through both the cell membrane and the nuclear membrane efficiently

**Figure 6:**
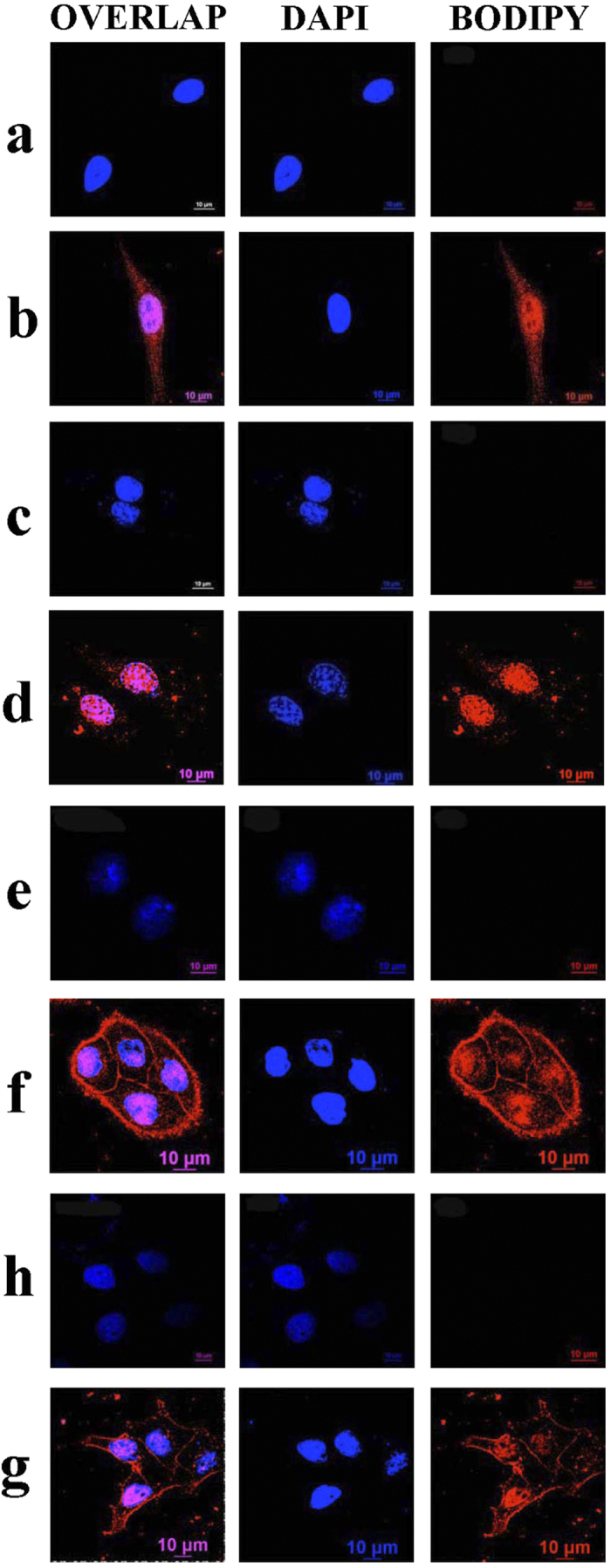
Membrane permibility of BODIPY and HFBI/BODIPY complex. BODIPY alone could not go inside the NIH-3T3 cell both at 4 h **(a)** and 24 h **(c).** HFBI/BODIPY complex could pass through both the cell membrane and the nuclear envople of NIH-3T3 cell at 4 h **(b)** and 24 h **(d)**. BODIPY alone could not go inside the HeLa cell both at 4 h **(e)** and 24 h **(g).** HFBI/BODIPY complex could pass through both the cell membrane and the nuclear envople of HeLa cell, and this complex slightly accmulated in or near the plasma membrane region only in HeLa cells at 4 h **(f)** and 24 h **(h)**.

From the above experiments, we demonstrated that HFBI-functionalized BODIPY obtained an excellent water-solubility due to its surface modification by HFBI. Since cell permeability is an essential requirement for the fluorescent probes to be used in the cellular environment, the membrane permibility of the HFBI/BODIPY complex was examined in the following. As shown in Fig. 6a, when the BODIPY dissolved in DMSO was applied into the NIH 3T3 cell, there was no fluorescence inside the cell at all after 4 h incubation. This results showed our BODIPY derivative could not pass through the cell membrane. However, when the BODIPY was modified with HFBI, the fluorescence was very srong and was spread out through the entir cell (Fig. 6b). Interestingly, it was notable that at least 50% of fluorescence signal was deteced in the nuclear zone. This observation suggested that some of HFBI/BODIPY complexes had the ability to move across the nuclear envelop. After 24 h, BODIPY dissolved in DMSO still could not get into the cell indicating its intricic property of low membrane permibility (Fig. 6c). However, it was clearly that HFBI-functionalized BODIPY still preserved strong fluorescence. Moreover, it seems like more and more HFBI/BODIPY complexes accumulated into the nuclear region of the cell after 24 h incubation (Fig. 6d). This result suggested there mighe be a dynamic relocation process after HFBI-functionalized BODIPY broke through both the cell and nuclear membranes. HeLa cell was also used to verify membrane permibility of the HFBI/BODIPY complex. In the control experiment, BODIPY itself could not pass though the cell membrane of the HeLa cell after at 4 h (Fig. 6e) and 24 h (Fig. 6g). These results were consistent with those ones obtained in NIH 3T3 cells, proving again our BODIPY dye naturally didn’t have the ability to penetrate the cell membrane barrier. Regarding to the HFBI-functionalized BODIPY complex, they showed strong fluorescence signals inside the HeLa cell both after 4 h (Fig. 6f) and 24 h (Fig. 6h). Similarly, the strongest fluorescent presented in the nuclear zone as well. However, there was also some difference about the BODIPY distribuation between HeLa and NIH 3T3 cell. For the HeLa cell, we could see clealy HFBI/BODIPY complex slightly accmulated in or near the plasma membrane region, but this accumulation was decreased at 24 h when compared with that at 4 h, implying part of HFBI/BODIPY complex might pass the cell membrane with slow rate. From the above results, we demonstrated that hydrophobin HFBI modification could render a high-membrane permibility of the BODIPY dye.

### HFBI/BODIPY complexes were nontoxic to NIH 3T3 and HeLa Cells

**Figure 7:**
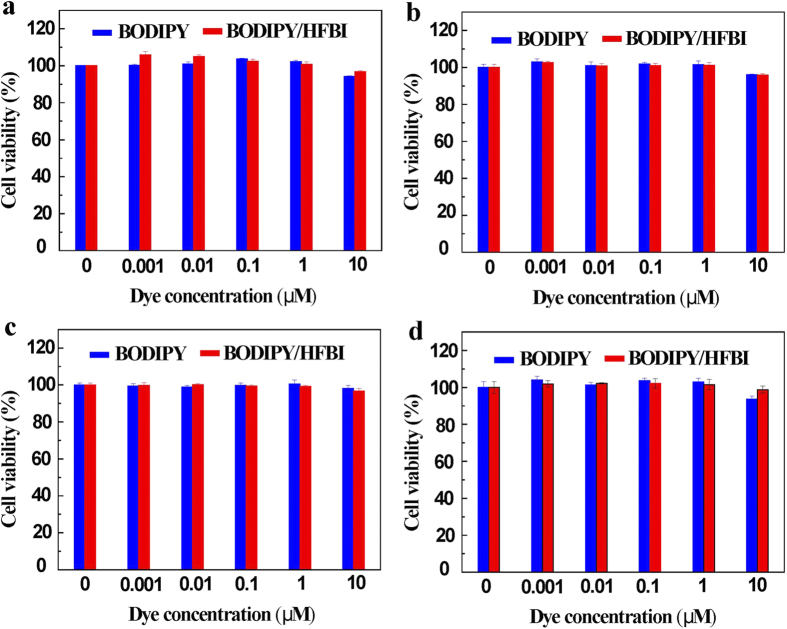
HFBI/BODIPY complexes were nontoxic to NIH-3T3 and HeLa Cells. **(a)** MTT assay showed that NIH-3T3 cell kept very high cell viability after exposure to different concentrations of HFBI/BODIPY complexes for 4 h. **(b)** NIH-3T3 cell obtained very high cell viability rate after 24 h exposure to HFBI/BODIPY complexes as well. **(c,d)** show HFBI/BODIPY complexes were nontoxic to HeLa cells at 4 h and 24 h respectively.

Extensive evidences have supported the nontoxic nature of hydrophobin coating on different interface or surfaces^40^. Specially, the surface hydrophobin layer has been proved to have a critical role in masking the immunogenicity of airborne fungal spores, implying the nontoxic property of hydrophobin-covered materials. According to our finding, we speculated HFBI/BODIPY complex was nontoxic to the cell that it interacted with, because of the coverage of HFBI film on the surface of BODIPY. In our real assays, the cell viabilities of NIH 3T3 and HeLa cells were determined upon exposure to different concentrations of HFBI/BODIPY complex (from 0.001 to 10 μM). As shown in Fig. 7, the administration of HFBI-functionalized BODIPY did not affect the cellular viability as compared with the control cells, even after prolonged exposure (48 h). This result indicated the cytotoxicity of HFBI/BODIPY complex toward NIH 3T3 and HeLa cells was very low. Therefore, HFBI-functionalized BODIPY can be applied in the living cell system for bio-imaging in the future.

## Discussion

In this paper, we showed a simple and reliable strategy for producing water-soluble and membrane permeable BODIPY dye by self-assembled hydrophobin. In our study, we found that BODIPY formed spherical particles in hydrophobin solution in a concentration dependent manner. Then we proved that spherical HFBI/BODIPY complexes were resulted from detaching from huge BODIPY aggregates by making “small hole” on their surfaces after sonication. Moreover, we demonstrated nontoxic hydrophobin-functionalized BODIPY could not only break through the cell membrane, but also the nuclear envelop efficiently. There were four advantages about our strategy to mofidy BODIPY dye according to our results. The first one was our method was rather simple and straightforward. Just simple mixing and sonication for 30 min could readily form HFBI/BODIPY complex. The second one was HFBI-functionalized BODIPY showed broad intracellular distribution. In general, the BODIPY dye had the tendence to accumulate into the subcellular memebrane due to its high lipophilicity property. Several BODIPY derivatives were reported to specially localize in the endoplasmic reticulum (ER) and Mitochondria^41^,^42^. Therefore, when people aim to image biological targets in the cytoplasm or in other specific organelles with a minimum non-specific background, hydrophilic groups are needed to introduce into BODIPY dyes by covalent modification in general^25^. Since HFBI-functionalized BODIPY could distribute throughout the whole cytoplasm, therefore, it was very easy to visualize specific biomolecules or organelles in the cell by linking a targeting group to the HFBI protein. The third one was that HFBI-functionalized BODIPY could pass through the nuclear envelop. To our knowledge, this was the first demonstration that BODIPY could localize in the nucleus. We believed that the size of the HFBI/BODIPY complex was contributed to this interesting observation. Enough small dimension could faciliate those round particles move across the nucler pore. By using HFBI-functionalized BODIPY, we may image the specific biological targets or drugs in the nucleus in the future. Finally, because HFBI protein presents numerous reactive groups such as hydroxyls, amines, thiols, carboxylic acids, and others, it will provide active sites for easy further surface modification of the BODIPY dyes and direct interaction with other biomolecules in specific ways that can be engineered. In summary, our strategy is the first demonstration of functionalization of fluorescent BODIPY dyes with amphiphilic protein hydrophobin, indicating the great potential of modification and functionalization chemical fluorescent dyes with biological molecules to open or broaden their applications.

## Methods

### Synthesis and characterizations of the BODIPY derivative

#### General

All solvents and starting materials were commercially available and were used without further purification (unless specially mentioned). Silica gel for column chromatography (CC) was 300–400 mesh. ^1^H NMR spectra were recorded on a Bruker AV400 MHz spectrometer in CDCl_3_ or with tetramethylsilane as a reference. MALDI-TOF-MS spectra were determined with a Bruker Autoflex TOF/TOF III instrument. The UV-Vis spectra of dyes in CH_2_Cl_2_ solution (3×10^−6^ M) were measured using Shimadzu UV-1800 in 10 mm quartz cell Spectrometer.

#### BDP

2,4-dimethylpyrrole (2.5 g, 26.6 mmol) was dissolved in HCl aqueous solution (0.12 mol/L, 300 mL) above ice-water bath, and methyl 4-formylbenzoate (2.1 g, 13.0 mmol) in ethanol was added dropwise. The reaction mixture was stirred for half an hour. Then the crude (4-methoxycarbonyl) phenyl-dipyrromethane (3.2 g) was obtained by drying the filter cake in a vacuum oven. Crude (4-methoxycarbonyl) phenyl-dipyrromethane (3.2 g) was dissolved in anhydrous CH_2_Cl_2_ (50 mL), and DDQ (1.4 g, 6.2 mmol) dissolved in a solvent of anhydrous THF (10 mL) was added dropwise. The mixture was stirred for 1 h. Then, triethylamine and BF_3_·Et_2_O were added dropwise into the mixture, and the mixture was stirred for 3 h in dark at room temperature. The reaction mixture was diluted with CH_2_Cl_2_ (50 mL) and washed three times with water, dried (Na_2_SO_4_) and evaporated prior to purification using chromatography (silica, CH_2_Cl_2_/hexane, 1:2) to afford the desired product as a red solid (1.8 g, 37%). ^1^H-NMR (400 MHz, CDCl_3_): δ 8.18 (d, J = 8.0 Hz, 2H), 7.41 (d, J = 8.0 Hz, 2H), 5.99 (s, 2H), 3.97 (s, 3H), 2.56 (s, 6H), 1.36 (s, 6H). MALDI-TOF-MS: [M]^+^ calcd for C_21_H_21_BF_2_N_2_O_2_, 382.1660, found 382.30.

#### TM

BDP (50 mg, 0.13 mmol) and N-decyl carbazole (132 mg, 0.39 mmol) were added to a 25 ml round bottomed flask containing 5 ml of toluene. Acetic acid (0.1 mL) and piperidine (0.1 mL) were added to the reaction mixture at the same time. The mixture was heated to 90 ^o^C and stirred 6 h. Then the mixture was extracted with DCM. The solvent was evaporated, dried (Na_2_SO_4_) and the residue was chromatographed on silica gel with hexanes: CH_2_Cl_2_ (1:2) as the eluent to afford the desired product as a blue solid (85.20 mg, yield 46.5%). ^1^H-NMR (400 MHz, CDCl_3_): δ 8.37 (s, 8.37 Hz, 2H), 8.23 (d, J = 7.7 Hz,4 H), 7.86 (4 m, 7.86 Hz, 4H), 7.52 (m, J = 13.8 Hz 6H), 7.46 (m, J = 8.2 Hz, 4H), 7.32 (t, J = 7.4 Hz, 2H), 6.74 (s, 2H), 4.34 (t, J = 7.1 Hz, 4H), 4.02 (s, 3H), 1.90 (m, 4H), 1.46 (s, 6H), 1.1–1.34 (m, 28H), 0.87 (t, 6H). MALDI-TOF-MS: [M]^+^ calcd for C_67_H_77_BF_2_N_4_O_2_, 1016.5951, found 1016.43.

### Preparation of HFBI/BODIPY complex

Four concentrations of HFBI solution (50, 100, 150, 200 μg/mL) were prepared by using phosphate buffers (pH 6.4). 1 mg of BODIPY was dissolved in 1 mL of DMSO and then 100 μL of BODIPY solution was added to four concentrations of HFBI respectively. The resulting mixtures were dispersed by ultrasonic agitation (120 W) for 30 min in iced water, and then the resulting suspension was subjected to ultra-centrifugation at 10000 rpm for 30 min at 4 ^o^C. Finally, 80% of the supernatant was collected and lyophilized for further analysis.

### Measurements

TEM images were obtained by field-emission TEM at 200 kV (JEM-2100F, JEOL, Japan) and SEM images were obtained by field-emission SEM at 3 kV (S-4800, Hitachi, Japan). The chemical compositions of BODIPY and HFBI/BODIPY were analyzed using XPS Apparatus (PHI-5300). The experiment conditions were as follows: the energy of excitation source monochromatic Mg-Kα radiation was 1253.6 ev, and the survey scan range was 0–1100 ev. Fourier-transform infrared (FTIR) spectra were obtained on a BRUKER IFS 55 FTIR system using the KBr disk method. The transmittance spectra were recorded at a resolution of 2 cm^−1^ between 4000 and 400 cm^−1^.

### Fluorescent staining of cells

NIH-3T3 and HeLa cells were grown separately on glass cover slips in 48-well plates with a seeding concentration of 1×10^4^ cells/well for 24 h. After that, cells were added with six different concentrations (0, 0.001, 0.01, 0.1, 1, 10 μM) of BODIPY and HFBI/BODIPY, and then cultured for both 4 h and 24 h. Afterwards, the plates were washed with 100 μL of PBS per well three times to remove probes which were still in the DMEM medium. Before confocal microscope (UltraVIEW VoX, PerkinElmer) imaging, all the samples were stained with DAPI for 5 min and washed with 100 μL of PBS per well three times. The excitation wavelength for BODIPY and HFBI/BODIPY was 633 nm.

### Cytotoxicity measurement

Cell viability measurements were performed by MTT analysis. NIH-3T3 and HeLa (1 × 10^5^ cells per well) cells were cultured in DMEM medium with 10% fetal bovine serum. Cells were seeded in 96-well flat-bottomed plates and incubated for 24 h at 37 ^o^C under 5% CO_2_. After 24 h of cell attachment, the plates were washed with 100 μL of PBS per well. The cells were then cultured in a medium with six different concentrations (0, 0.001, 0.01, 0.1, 1, 10 μM) of BODIPY and HFBI/BODIPY for 48 h. Cells in a culture medium without fluorescent dyes were used as control. MTT (10 μL, 5 mg mL^−1^) in PBS was subsequently added to each well. The plates were then incubated at 37 ^o^C for 4 h in a 5% CO_2_ humidified incubator. The medium was carefully removed, and the products were lysed in 200 μL of DMSO. The plate was shaken for 10 min, and the absorbance was measured at 490 nm and 570 nm using a microplate reader (EnSpire Multilabel Reader, PerkinElmer).

## Additional Information

**How to cite this article**: Wang, K. *et al*. Self-assembled hydrophobin for producing water-soluble and membrane permeable fluorescent dye. *Sci. Rep.* **6**, 23061; doi: 10.1038/srep23061 (2016).

## Supplementary Material

Supplementary Information
