# Supplementary material for: Self-assembled hydrophobin for producing water-soluble and membrane permeable fluorescent dye
Source: Sci Rep. 2016 Mar 15;6:23061. doi: 10.1038/srep23061 (PMC4791660; doi:10.1038/srep23061)
Supplement: Supplementary Information [file srep23061-s1.pdf]

Self-assembled hydrophobin for producing water-soluble and membrane permeable fluorescent dye

Kunpeng Wang [1, #], Yunjie Xiao [2, 3, #], Yanyan Wang [1], Yaqing Feng [1, 4], Cheng Chen [1], Jie Zhang [1], Qian Zhang [1], Shuxian Meng [1, *], Zefang Wang [1, 2, 3, *] & Haitao Yang [1]

[1]*School of Chemical Engineering and Technology, School of Life Sciences, College of Precision Instrument and Opto-electronics Engineering, Tianjin University, Tianjin 300072, People's Republic of China.*

[2]*State Key Laboratory of Medicinal Chemical Biology, College of Pharmacy, Nankai University, Tianjin 300071, People's Republic of China.*

[3]*Tianjin International Joint Academy of Biotechnology and Medicine, Tianjin 300457, People's Republic of China.*

[4]*Collaborative Innovation Center of Chemical Science and Engineering, Tianjin 300072, People's Republic of China*

[#]These authors contributed equally to this work.

*Correspondence and requests for materials should be addressed to Z.F.W. (zefangwang@tju.edu.cn) or to S.X. M. (msxmail@tju.edu.cn).

Supplementary Figure-1

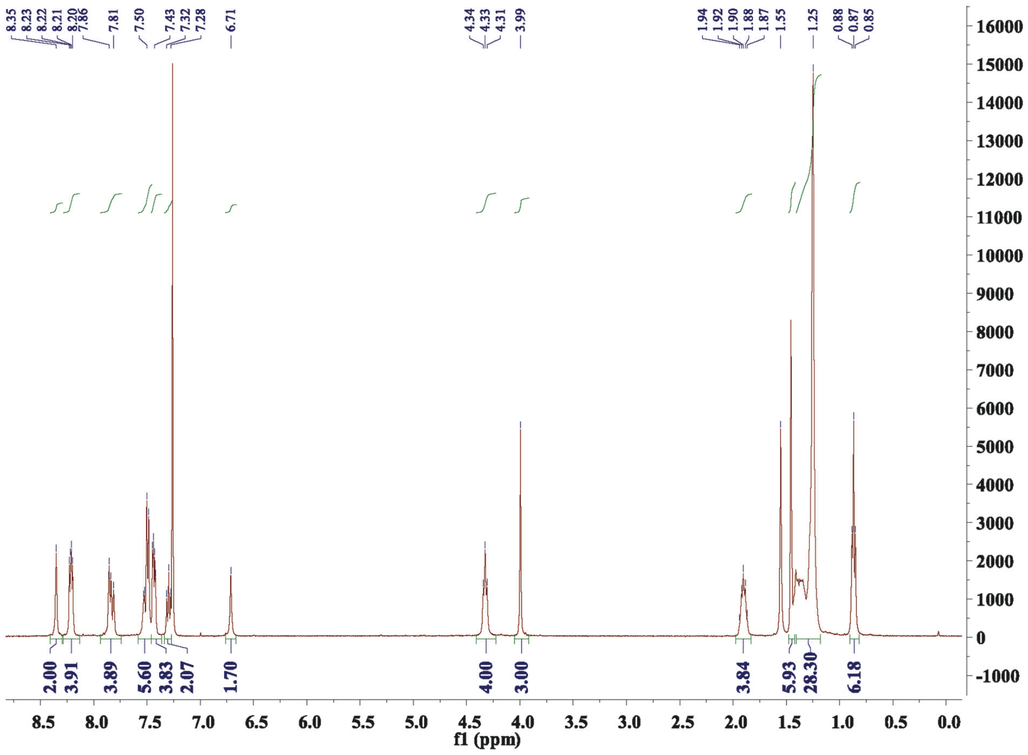

Figure 1. ^{1}H NMR of the BODIPY derivative.

Supplementary Figure-2

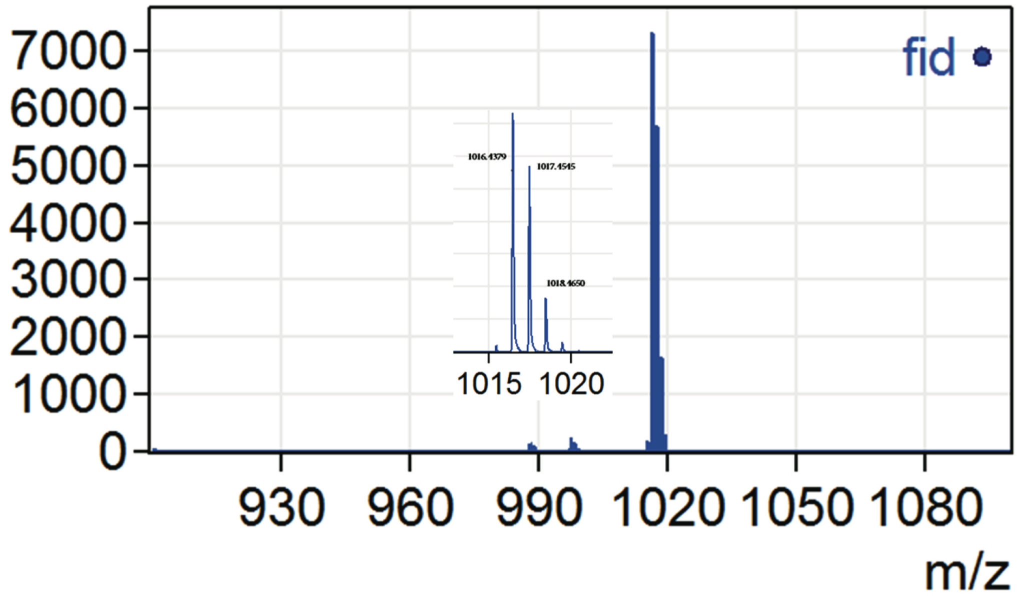

Figure 2. MALDI-TOF-MS of the BODIPY derivative.

Supplementary Figure-3

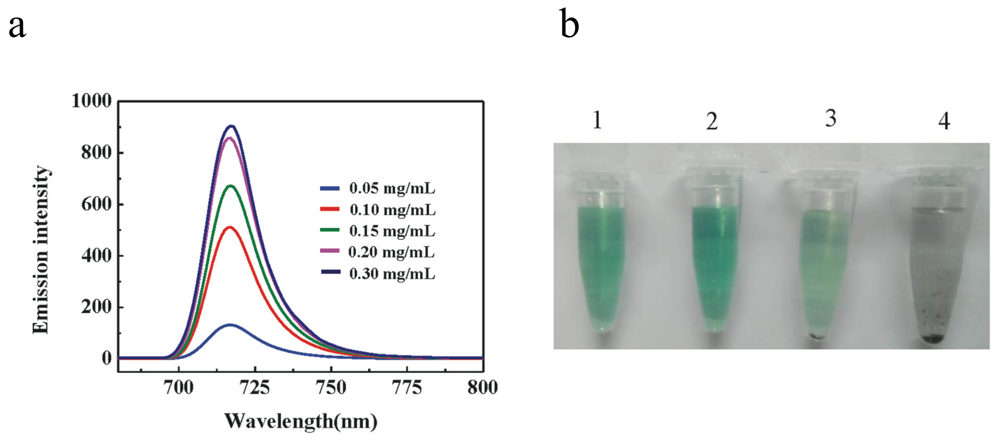

Figure 3. Hydrophobin concentration is a critical factor for the dispersion of the BODIPY dye. a. The fluorescence spectra of the same concentration of BODIPY solubilized at different concentrations (50, 100, 150, 200 and 300 μg/mL) of HFBI; b.1. BODIPI in 0.2 mg/mL HFBI, 2. BODIPI in 0.3 mg/mL HFBI, 3. BODIPI in 0.3 mg/mL HFBI (stand still within three weeks), 4. BODIPY in pure water.
